# Aphicidal Potential and Metabolomic Profiling of Floriculture Leaf Waste Extracts Against *Aphis craccivora* Koch Under Laboratory Conditions

**DOI:** 10.1002/cbdv.71720

**Published:** 2026-09-30

**Authors:** Aditya Singh Ranout, Rahul Kumar, Rupinder Kaur, Anish Kaachra, Anil Kumar, Dinesh Kumar, Bhavya Bhargava, Gireesh Nadda

**Affiliations:** ^1^ Entomology Laboratory Agrotechnology Division CSIR‐Institute of Himalayan Bioresource Technology (IHBT) Palampur Himachal Pradesh India; ^2^ Academy of Scientific and Innovative Research (AcSIR) Ghaziabad India; ^3^ Fermentation and Phytofarming Technology Division CSIR‐Institute of Himalayan Bioresource Technology (IHBT) Palampur Himachal Pradesh India; ^4^ Chemical Technology Division CSIR‐Institute of Himalayan Bioresource Technology (IHBT) Palampur Himachal Pradesh India; ^5^ Floriculture Laboratory Agrotechnology Division CSIR‐Institute of Himalayan Bioresource Technology (IHBT) Palampur Himachal Pradesh India

**Keywords:** biopesticides, cowpea aphid, floriculture, metabolomics, wastes

## Abstract

Farmers are shifting their interest from conventional to floriculture crops. The leaves of floriculture crops remain unused and are often discarded as waste. These wastes are a rich source of industrially valuable compounds with diverse biological activities. In this study, the methanolic extracts of leaf wastes of three floriculture crops, namely, *Chrysanthemum grandiflorum*, *Tagetes patula*, and *Alstroemeria hybrida*, were studied for their insecticidal potential against nymphs and adults of *Aphis craccivora*. The results of the study showed that *C. grandiflorum* extract (LC_50_ = 637.49 ppm) exhibited higher insecticidal potential, followed by the extracts of *T. patula* and *A. hybrida* against adult *A. craccivora*. Similarly, nymphicidal bioassays showed that *C. grandiflorum* extract (LC_50_ = 1398.36 ppm) was the most active, followed by *T. patula* and *A. hybrida*. The targeted and nontargeted metabolomics revealed the presence of insecticidal metabolites in the studied extracts. Enzyme activity studies of *C. grandiflorum* extracts showed acetylcholinesterase (AChE) inhibition activity, whereas *T. patula* and *A. hybrida* extracts showed AChE induction. Moreover, the studied extracts showed glutathione‐S‐transferase (GST) induction, with *C. grandiflorum* showing the most, followed by *A. hybrida* and *T. patula* extracts. This study advances the field of waste‐to‐wealth by utilizing the studied leaf wastes for the development of biopesticides.

## Graphical abstract

We utilized the leaf wastes of floriculture crops, namely *Tagetes patula*, *Chrysanthemum grandiflorum*, and *Alstroemeria hybrida* to determine their insecticidal potential against nymphs and adults of *Aphis craccivora*. Targeted and nontargeted metabolomics of extracts showed the presence of phenols and flavonoids, which corroborates the insecticidal activity.

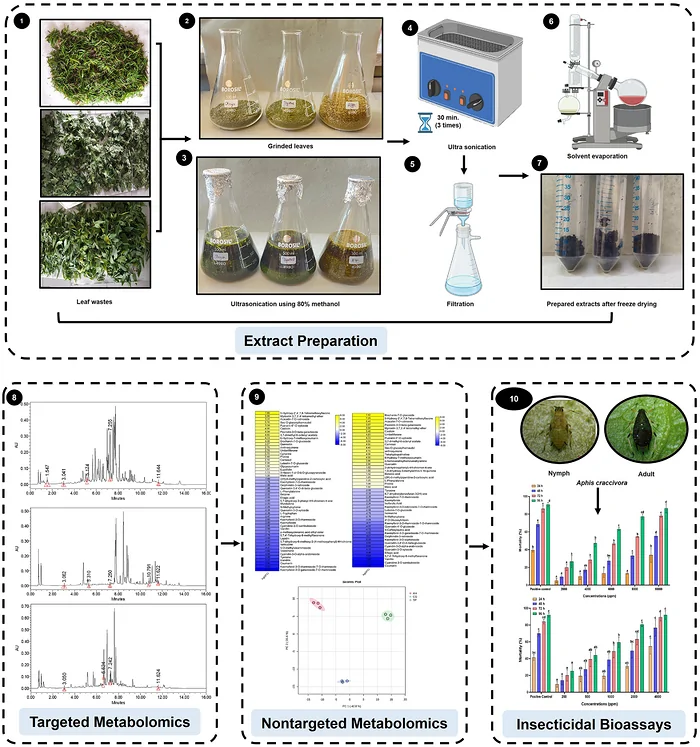

## 1 Introduction

*Aphis craccivora* Koch (Hemiptera: Aphididae) is the most damaging, polyphagous, and cosmopolitan pest that infests approximately nineteen plant families in greenhouse and open agricultural landscapes [1]. *A. craccivora* (cowpea aphid) damaged the crops, directly by sucking the host plant sap, which is crucial for plant growth, resulting in stem wilting, leaf curling, and ultimately leading to the death of the host plant. Indirectly, it is a vector of many plant viruses like bean common mosaic virus (BCMV), cucumber mosaic virus (CMV), alfalfa leaf curl virus (ALCV), and papaya ring spot virus (PRSV), which significantly reduces crop production [2, 3]. During severe infestations, they secrete honeydew (sticky substance) which further attracts the growth of sooty mold. Further, these sooty molds disturb photosynthesis and lead to the retarded growth of plants and ultimately reduce crop production. The management of *A. craccivora* is very challenging due to its short life cycle, high fecundity rate, and polyphagous nature [4, 5, 6].

Chemical control strategies are primarily used to manage aphid populations in the greenhouse and open agricultural landscapes. Mainly, broad‐spectrum insecticides like carbamates, organophosphates, pyrethroids, and so forth are commonly used [7]. Dependence on synthetic insecticides leads to insect resurgence, resistance, increased pesticide residues, soil deterioration and also causes harm to nontargeted organisms and humans too [8]. Therefore, there is an urgent need to develop environmentally friendly and effective methods to control aphid populations and prevent the damage induced by synthetic insecticides on the environment. The use of plant‐derived biopesticides presents a promising alternative for managing aphid populations due to their diverse modes of action, including stomach poisons, antifeedants, repellents, nerve poisons, contact poisons, and growth inhibitors [9, 10]. Extracts, fractions, and molecules derived from various plants, including *Achyranthes bidentata* and *Rosmarinus officinalis* have demonstrated insecticidal potential against *A. craccivora* [11, 12].

The Indian floriculture market size was valued at INR 323.8 billion in 2025 and is projected to reach INR 793.1 billion by 2034 with the CAGR value of 10.5% [13]. Consequently, the floriculture industry is emerging as a diverse and significant option, offering benefits from aesthetic, social, environmental, and economic perspectives. Recently, this sector has become a lucrative agribusiness, driven by the rising demand for floricultural products, which can be attributed to enhanced living standards and a growing awareness among individuals about the importance of an eco‐friendly environment [14]. The profitability of the business presents a significant challenge for farmers due to leftover materials; however, these leftovers are a rich source of valuable metabolites that can be investigated for biopesticide development. As a zero‐cost and readily available biomass, floriculture wastes serve as an ideal resource than medicinal plants. Floriculture crops like *Chrysanthemum* spp. and *Tagetes* spp. have demonstrated insecticidal potential as a sustainable and eco‐friendly alternative for the management of *A. craccivora* [15]. Although no studies have been reported on the insecticidal potential of the *Chrysanthemum grandiflorum*, *Tagetes patula*, and *Alstroemeria hybrida* leaf wastes against *A. craccivora*.

Keeping these research gaps in mind, the present study examines the necessity for eco‐friendly alternatives to manage *A. craccivora* population while addressing the floriculture waste problems. This study focuses on determining the insecticidal potential of leaf waste generated from three specific floriculture crops: *C. grandiflorum*, *T. patula*, and *A. hybrida* against nymphs and adults of *A. craccivora*. This study also explored the metabolic profiles of methanolic extracts from *C. grandiflorum*, *T. patula*, and *A. hybrida* using both targeted and nontargeted metabolomic approaches. Additionally, this study evaluated the enzyme‐inhibitory effects of methanolic extracts from the leaf waste of the studied floriculture crops to elucidate their mode of action.

## 2 Materials and Methods

### 2.1 Chemicals and Reagents

The chemicals and reagents utilized in the studies were of high‐performance liquid chromatography (HPLC) grade quality. Methanol, acetonitrile, formic acid, triton‐X, and standards (purity ≥ 98%) were sourced from Sigma–Aldrich, India. Neem Baan was obtained from Rentokil‐PCI Pvt. Ltd., India. Bradford reagent was obtained from Himedia Pvt. Ltd. in India. Kits for determining the activity of acetylcholinesterase (AChE) and glutathione‐S‐transferase (GST) were obtained from Abcam, United Kingdom.

### 2.2 Collection and Extract Preparation of Floriculture Crop's Leaf Wastes

Leaves of different floriculture crops, namely *C. grandiflorum*, *T. patula*, and *A. hybrida* were collected from the fields of the Floriculture Department, Agrotechnology Division, CSIR‐IHBT Palampur, on 16^th^ August, 2024. Specimens of *C. grandiflorum*, *T. patula*, and *A. hybrida* were deposited in the CSIR‐IHBT herbarium with voucher nos. PLP28702, PLP28701, and PLP28703, respectively. Specimens were identified by Dr. Vikas Kumar (Scientist D, CSIR‐IHBT). Collected leaves of these crops were shade‐dried to constant weight under ambient conditions. Dried leaves of the respective plants were ground into fine powder. 100 g of ground leaves of individual plants were subjected to a four‐step methanol:water (80:20) extraction due to its ability to extract intermediate polarity metabolites, including targeted polyphenols and lipophilic polymethoxylated flavones. Briefly, 400 mL of methanol (80%) was poured into each conical flask containing ground leaves of *C*. *grandiflorum*, *T. patula*, and *A. hybrida* followed by ultrasonic bath sonication (37 kHz) for 30 min. After sonication, solvent with extracted metabolites obtained from different leaf wastes was filtered using filter paper (Whatman no. 1) separately. Consequently, a similar process was repeated thrice with 100 mL of solvent to get 700 mL of liquid extracts of leaf wastes of each plant. The obtained liquid extracts were evaporated under reduced pressure at 40°C followed by freeze‐drying, and stored at −20°C, separately for chemical characterization and evaluation of their insecticidal potential. The percent yields of the prepared methanolic extracts were calculated using the following formula:

$$\mathrm{Extract}\;\mathrm{Yield}\;\left(\%\right)=\frac{\mathrm{Weight}\;\mathrm{of}\;\mathrm{dried}\;\mathrm{extract}\;\left(g\right)}{\mathrm{Weight}\;\mathrm{of}\;\mathrm{dried}\;\mathrm{leaf}\;\mathrm{wastes}\;\left(g\right)}\;\times 100$$

### 2.3 Rearing and Maintenance of *Aphis craccivora*

Aphids were reared and maintained in insect cages on lab‐grown two‐week‐old *Phaseolus vulgaris* (common bean) plants. These aphids were reared and maintained in the insectary of the Entomology Laboratory at CSIR‐Institute of Himalayan Bioresource Technology, India for over 100 generations, with no prior exposure to pesticides. The rearing conditions of the insectary were maintained at a temperature of 25°C ± 2°C, 60% ± 10% relative humidity (RH), and a photoperiod of 16: 8 (L: D). Nymphs (second instar) and adults (1 day old) were used to evaluate the aphicidal potential of the prepared extracts.

### 2.4 UPLC‐PDA‐Based Analysis of Phenolic Acids in Leaf Wastes of Floriculture Crops

Prepared extracts of leaf wastes were dissolved in methanol (LC–MS grade) to make the final concentration of 10 mg/mL, followed by filtration with a 0.22 µm PTFE syringe filter. The samples were then used for UPLC‐PDA analyses. Standards of selected phenolic acids flavonoids' were used for the qualitative and quantitative analysis. Stock solutions of standards (1 mg/mL) were dissolved in methanol (LC–MS grade) and were also filtered through a 0.22 µm filter. The calibration curve was made by serial dilution of stock solution ranging from 2 to 200 ppm. Standard identification on UPLC‐PDA was done based on UV wavelength and retention time (RT), while quantification was done by plotting the calibration curve and concentration was calculated using area under the curve. Waters Acquity UPLC, an H‐class system equipped with an ACE Ultracore SuperC18 column (2.1 mm X 100 mm, 2.5 µm), was used for the quantification of selected phenolic acids and flavonoids at 270 nm. The gradient elution system having mobile phase A (0.1% formic acid in water) and mobile phase B (0.1% formic acid in acetonitrile) was used for the efficient separation of metabolites. The gradient started from 0 min at 5% B, maintained till 0.3 min; the concentration of B increased to 30% from 0.3 min to 9 min, then 30% B to 70% B; 9–11 min, 50% B; from 11 to 12 min, 50% B, then at 12.2 min, the mobile phase was maintained to initial conditions, 5% B, maintained till 16 min [16]. Elution was performed at a solvent flow rate of 0.30 mL/min. This UPLC‐PDA method was validated as per ICH (Q2/R1) guidelines. All parameters were tested as mentioned in the guidelines, that is, linearity, limit of detection, limit of quantification, accuracy by recovery analysis, and precision by interday and intraday analysis. Quantification of compounds present in the samples was done by plotting the calibration curve and area of the peak, measured on the basis of matching the RT and UV spectra.

### 2.5 Nontargeted Metabolomics Using UHPLC‐QTOF‐MS/MS

The nontargeted metabolomics of leaf waste extracts of the studied floriculture crops were carried out using the ion mobility LC–MS (Agilent, USA). For chromatographic separation, the Acquity BEH C18 column (2.1 mm × 100 mm, 1.7 µm) was used using mobile phase A (0.1% formic acid in water) and mobile phase B (0.1% formic acid in acetonitrile). The gradient system consisted of 5% B at 0 min, further increased from 5% to 20% till 4 min, then 20%–30% B for 4 to 6 min, 40% B from 6 to 10 min, 50% B from 10 to 12 min, and 58% B from 12 to 18 min. The mobile phase concentration was then decreased to 25% from 18 to 22 min, followed by the restoration to the initial condition of 5% B at 25 min. Elution was performed at a solvent flow rate of 0.25 mL/min. Mass chromatograms were processed and annotated with MS‐DIAL [17, 18]. Briefly, raw data files were transformed into ABF format for compatibility with MS‐DIAL. The parameters utilized for data processing included a mass tolerance of 0.05 Da, a RT tolerance of 0.5 min, an identification score cutoff of 85%, and a relative abundance cutoff of 0.5%. For metabolite identification, the query LC–MS data, includes RT, MS, and MS–MS spectra, were aligned with reference metabolites in the curated spectral library [18]. Furthermore, the data were normalized by aligning values to the median to facilitate comparison across samples and reduce inter‐sample variability.

### 2.6 Insecticidal Bioassays Against Nymphs and Adults of *Aphis craccivora*

Nymphs and adults of *A. craccivora* of uniform age were subjected to the evaluation of the insecticidal potential of prepared extracts of leaf wastes from *T. patula*, *C*. *grandiflorum*, and *A. hybrida*. Different test concentrations of extracts were prepared by dissolving prepared extracts in 0.01% Triton X. The bioactivity of extracts was evaluated by the Leaf Disc Direct‐Potter Tower Method. Briefly, 10 mL of 1.5% Agar solution was poured into each Petriplate (55 mm), and a *P. vulgaris* leaf disc (45 mm) was placed on semi‐solid agar with the abaxial (underside of the leaf) surface positioned upwards. The leaf was properly placed on the semi‐solid agar to ensure that the leaf remained hydrated throughout the experiment. Ten nymphs and adults of *A. craccivora* were released on the midrib of leaf discs and allowed to settle. Different test concentrations of methanolic extracts of *C. grandiflorum* (250–4000 ppm), *T. patula* (2000–10 000 ppm), and *A. hybrida* (5000–25 000 ppm) were used based on the preliminary range finding experiments. 1 mL of test concentrations of prepared extracts and 0.01% Triton X were used in treatment and control groups, respectively, using the Potter spray tower (Burkard Scientific, UK). The spray tower was operated at 68.9 kPa (10 psi) pressure. All experiments were conducted at the above‐mentioned rearing conditions. Each treatment with three replicates having 10 individuals each was repeated five times; that is, a total of 150 individuals were tested per concentration. Neem Baan (0.15% azardirachtin) at the recommended dose (2 mL/L) was used, corresponding to the manufacturer's recommended field dose as a positive control. The observations for insecticidal potential of test concentrations were recorded at 24, 48, 72, and 96 h after treatment (HAT). The immobile individuals who failed to react when nudged with a camel hairbrush were considered deceased.

### 2.7 Detoxification Enzyme Activity

#### 2.7.1 Preparation of Enzyme Solutions

The activities of detoxifying enzymes (AChE and GST) were assessed in adult *A. craccivora* that survived 24 and 48 h post exposure to the median lethal concentration (LC_50_) of each prepared extract, the negative control, and the recommended dosage of Neem Baan as a positive control. For every treatment, three replicates were used, and surviving *A. craccivora* adults were collected after 24 and 48 h of treatment. Aphids were weighed and homogenized in 0.1 M phosphate buffer saline (PBS) (1:9) on ice using a sterile pestle to maintain low temperatures and minimize protein/enzyme denaturation, followed by centrifugation at 12 000 rpm for 30 min at 4°C. After that, supernatants of each group were collected, labelled as their respective enzyme solution (ES), and stored at −20°C for detoxification enzyme studies.

#### 2.7.2 Determination of Protein Content in Enzyme Solution of Different Groups

The protein concentration of all ESs was estimated using the Bradford method [19]. Briefly, in a 96‐well microplate, 10 µL of prepared ES along with 30 µL of 0.1 M PBS and 160 µL Bradford reagent (Coomassie Brilliant Blue) was added. The reaction mixture was incubated at ambient temperature for 15 min. The absorbance was taken at 595 nm using a plate reader (BioTek Synergy H1). Bovine serum albumin (BSA) was used as a standard to estimate the protein concentration. Further, ES dilutions were prepared based on the lowest protein concentration estimated [12] for different groups.

#### 2.7.3 Determination of Acetylcholinesterase (AChE) and Glutathione‐S‐transferase Activity (GST)

For the detection of AChE activity in the prepared ES, the AChE Assay Kit (Colorimetric) Abcam ab138871 was used following the user's manual. Briefly, the wells comprising 25 µL diluted ES and 25 µL reaction mixture (50 µL DTNB, 50 µL acetylcholine, and 900 µL assay buffer) were incubated for 30 min at room temperature [11, 12]. Following incubation, AChE activity was measured at 410 nm using a microplate reader. The activity of the enzyme was expressed as micromoles per milliliter of protein per minute (µmol/ min/ mL protein).

For the estimation of GST enzyme activity, the GST Activity Assay Kit (Colorimetric) Abcam, ab65326 was used following the user's manual. Briefly, a 105 µL reaction mixture was prepared, having 10 µL diluted ES, and 40 µL assay buffer was added to the sample wells. In the case of control, 50 µL assay buffer was used. All the wells contain 5 µL glutathione and a 50 µL GST reaction mixture of GST Assay Buffer and GST Substrate (1‐chloro 2,4‐dinitrobenzene solution (CDNB) Solution (49:1)). The reaction mixture was mixed carefully, and the absorbance was measured at 340 nm in kinetic mode (every 2 min, for 30 min at 37°C). GST activity was calculated using the GS‐DNB extinction coefficient of 0.0096 µM^−1^cm^−1^ for CDNB [20]. Enzyme activity was expressed as micromoles per milliliter of protein per minute (µmol/ min/ mL protein). All the estimations were performed in triplicate.

### 2.8 Statistical Analyses

Corrected mortality was not calculated due to the absence of fatalities in the control group during nymphicidal and adulticidal bioassays [21]. The probit analysis [22] was used to calculate the lethal concentrations (LC_50_ and LC_90_) and lethal times (LT_50_ and LT_90_). Percent mortality and enzyme activity data were analyzed using one‐way analysis of variance (ANOVA) and subsequently evaluated using Duncan's test with *p* ≤ 0.05 using SPSS (IBM, Version 27.0.1) software. Graphs depicting percent mortality, spearman rank correlation coefficients (*ρ*) heat map, and enzyme activity were made using GraphPad Prism 8.0. For nontargeted metabolomics, the MetaboAnalyst 6.0 software was used to compute statistical analyses. Multivariate analyses of the data were employed after data normalization (median), log2 transformation, and autoscaling.

## 3 Results

### 3.1 Extract Yield of Leaf Wastes From Different Floriculture Crops

100 g of powdered leaves of *C. grandiflorum*, *T. patula*, and *A. hybrida* resulted in 12.76, 11.57, and 11.61 g of methanolic extract, respectively. The percent yields of the extract from leaf wastes of *C. grandiflorum*, *T. patula*, and *A. hybrida* were 12.76%, 11.57%, and 11.61%, respectively.

### 3.2 UPLC‐PDA‐Based Analysis of Phenolic Acids

The developed method was successfully validated in accordance with ICH Q2 (R1) guidelines. All acceptance criteria were met within the specified range. The detailed validation parameters and results are given in Table 1. The developed UPLC‐PDA method was further applied for the analysis of prepared extracts of leaf wastes used in this study. Polyphenols were identified and quantified in all methanolic extracts of the leaves from floriculture crops, including *T. patula*, *C. grandiflorum*, and *A. hybrida*, through UPLC‐PDA quantification (Figure S1). The results indicated that *T. patula* extract contains significant concentrations of gallic acid (21.51 ± 0.19 µg/10 mg) and ferulic acid (84.15 ± 0.34 µg/10 mg). In contrast, *C. grandiflorum* extract also contains gallic acid, but below the detection limits, although it does have trace amounts of ferulic acid (0.65 ± 0.00 µg/10 mg). Protocatechuic acid was found in significant quantities in the extracts of *T. patula* (7.85 ± 0.20 µg/10 mg) and *C. grandiflorum* (6.90 ± 0.32 µg/10 mg), while only trace amounts were detected in leaf wastes of *A. hybrida* (0.77 ± 0.00 µg/10 mg). The extract of *A. hybrida* is rich in ferulic acid as compared to other quantified phenolics (Table 2).

**TABLE 1:** Regression equations, linear ranges, LOD, LOQ, intraday, interday, and recovery study for floriculture leaf extracts.

Criteria	Gallic acid	Procatechuic acid	Vanillic acid	Syringic acid	Caffeic acid	EC	EGCG	*p*‐Coumaric acid	Ferulic acid	Rutin	Quercetin	Luteolin	Kaempferol
Retention time	1.82	3.16	5.66	6.02	6.38	6.63	7.78	8.19	8.41	9.15	11.96	12.01	13.21
Linear regression equation R^2^	*y* = 5763.9x – 473.79 *R* ^2^ = 0.999	*y* = 14365x + 1498.4 *R* ^2^ = 0.998	*y* = 17176x + 2171.8 *R* ^2^ = 0.9983	y = 20115x + 4359.5 R^2^ = 0.9978	*y* = 11277x + 1354.4 *R* ^2^ = 0.9985	*y* = 3155.5x + 712.72 *R* ^2^ = 0.9976	*y* = 5657.4x – 663.3 *R* ^2^ = 0.9986	*y* = 13995x + 167.12 *R* ^2^ = 0.9984	*y* = 11376x + 226.64 *R* ^2^ = 0.9987	*y* = 9577.6x – 371.78 *R* ^2^ = 0.9984	*y* = 22140x – 4223.8 *R* ^2^ = 0.9985	*y* = 6840.7x – 2407.3 *R* ^2^ = 0.9991	*y* = 19661x = 6693.3 *R* ^2^ = 0.9995
Inter day precision (% RSD)	0.311	0.423	0.050	0.023	0.066	0.021	0.045	0.017	0.025	0.023	0.012	0.018	0.019
Intraday precision (% RSD)	0.193	0.365	0.180	0.118	0.251	0.100	0.046	0.135	0.056	0.041	0.029	0.034	0.025
LOD (µg/mL)	0.75	1.12	0.80	0.20	0.26	0.26	0.50	0.39	0.72	0.48	0.61	1.22	0.59
LOQ (µg/mL)	2.28	3.39	2.43	0.62	0.79	0.78	1.52	1.17	2.18	1.46	1.86	3.69	1.78
Recovery (%)	99.48	99.08	91.60	92.83	103.87	103.38	92.31	95.23	104.77	98.01	91.21	98.83	99.56

**TABLE 2:** Phenolics profile (µg/10 mg) of methanolic extracts of leaf wastes of floriculture crops using UPLC‐PDA.

Plant	Gallic acid	Protocatechuic acid	Caffeic acid	*p‐*Coumaric acid	Ferulic acid	Luteolin	Kaempferol
*Tagetes patula*	21.51 ± 0.19	7.85 ± 0.20	0.14 ± 0.00	0.18 ± 0.00	84.15 ± 0.34	ND	0.08 ± 0.00
*Chrysanthemum grandiflorum*	ND	6.90 ± 0.32	0.22 ± 0.00	ND	0.65 ± 0.00	3.01 ± 0.02	NQ
*Alstroemeria hybrida*	ND	0.77 ± 0.00	ND	1.04 ± 0.03	12.17 ± 0.22	ND	NQ

Values represent the mean of phenolic compound ± standard deviation (*n* = 3).

*Abbreviations*: ND, not detected; NQ, not quantified.

### 3.3 Nontargeted Metabolomics Using UHPLC‐QTOF‐MS/MS

A total of 76 metabolites were annotated upon alignment with the reference library using MS‐DIAL post UHPLC‐QTOF‐MS/MS (Table S1). Furthermore, median‐aligned normalized values (Figure S2) illustrate the central tendency of each group relative to the median. Principal component analysis revealed variation among metabolites in the leaf wastes of selected floriculture crops. The findings indicated that the first principal component (PC1) showed 46.7% of the total variance in the dataset, whereas the second principal component (PC2) accounted for an additional 34.7% of the overall variance (Figure 1). The two components accounted for a cumulative variance of 81.4% across all data points, highlighting the variations in the analyzed data. Additionally, all three extract datasets resulted in distinct groups, differentiating them from one another (Figure 1). Out of 76 metabolites, 36 were selected for the relative accumulation across the leaf extracts of *C. grandiflorum* and *A. hybrida*. The fold change analysis showed that 15 metabolites were highly accumulated in *C. grandiflorum* extract as compared to *A. hybrida* (Figure 2A). Among them, acacetin‐7‐O‐rutinoside (5.31), quercetin (3.57), umbelliferone (2.78), carnosol (2.10), and luteolin‐7‐O‐glucoside (2.08) were accumulated in higher quantities (Figure 2A). On the other hand, 21 metabolites such as cyanidin‐3‐O‐alpha‐arabinoside (−4.85), cyanidine‐3‐O‐sambubioside (−2.96), quercetin‐3‐O‐xyloside (−2.34), and delphinidin‐3‐rutinoside (−1.96) showed lower accumulation in *C. grandiflorum* extract as compare to the *A. hybrida* (Figure 2B). Furthermore, comparative fold change analysis of the metabolites in *C. grandiflorum* and *T. patula* leaf extracts showed that 13 metabolites, including acacetin‐7‐O‐rutinoside (6.40), umbelliferone (4.81), anthraquinone (4.15), and quercetin (2.94), showed higher accumulation in *C. grandiflorum* extract (Figure 2B). Conversely, 20 metabolites, such as coumarin (−7.88), vanillin (−7.46), quercetin‐3‐O‐xyloside (−6.12), kaempferol 3‐O‐sophoroside (−5.59), and isoferulic acid (−2.34), showed lower accumulation in *C. grandiflorum* as compare to *T. patula* leaf extract (Figure 2B).

**FIGURE 1:**
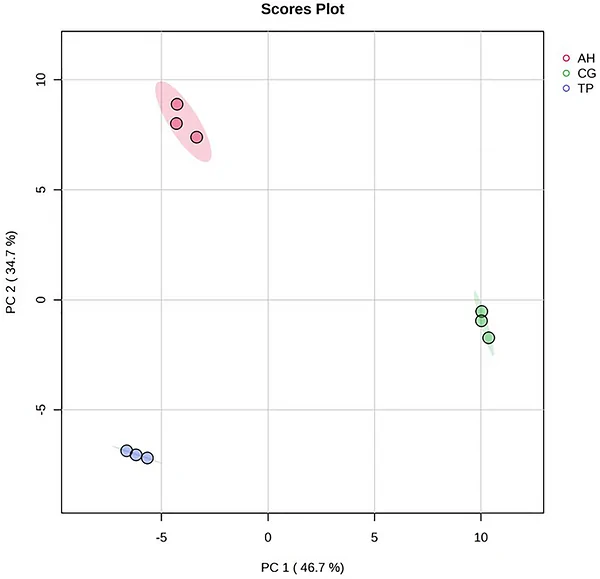
Principal component analysis (PCA) score plot based on Spearman correlation of the detected metabolites in *Tagetes patula* (TP), *Chrysanthemum grandiflorum* (CG), and *Alstroemeria hybrida* (AH) leaf extracts. Each point represents an individual biological replicate.

**FIGURE 2:**
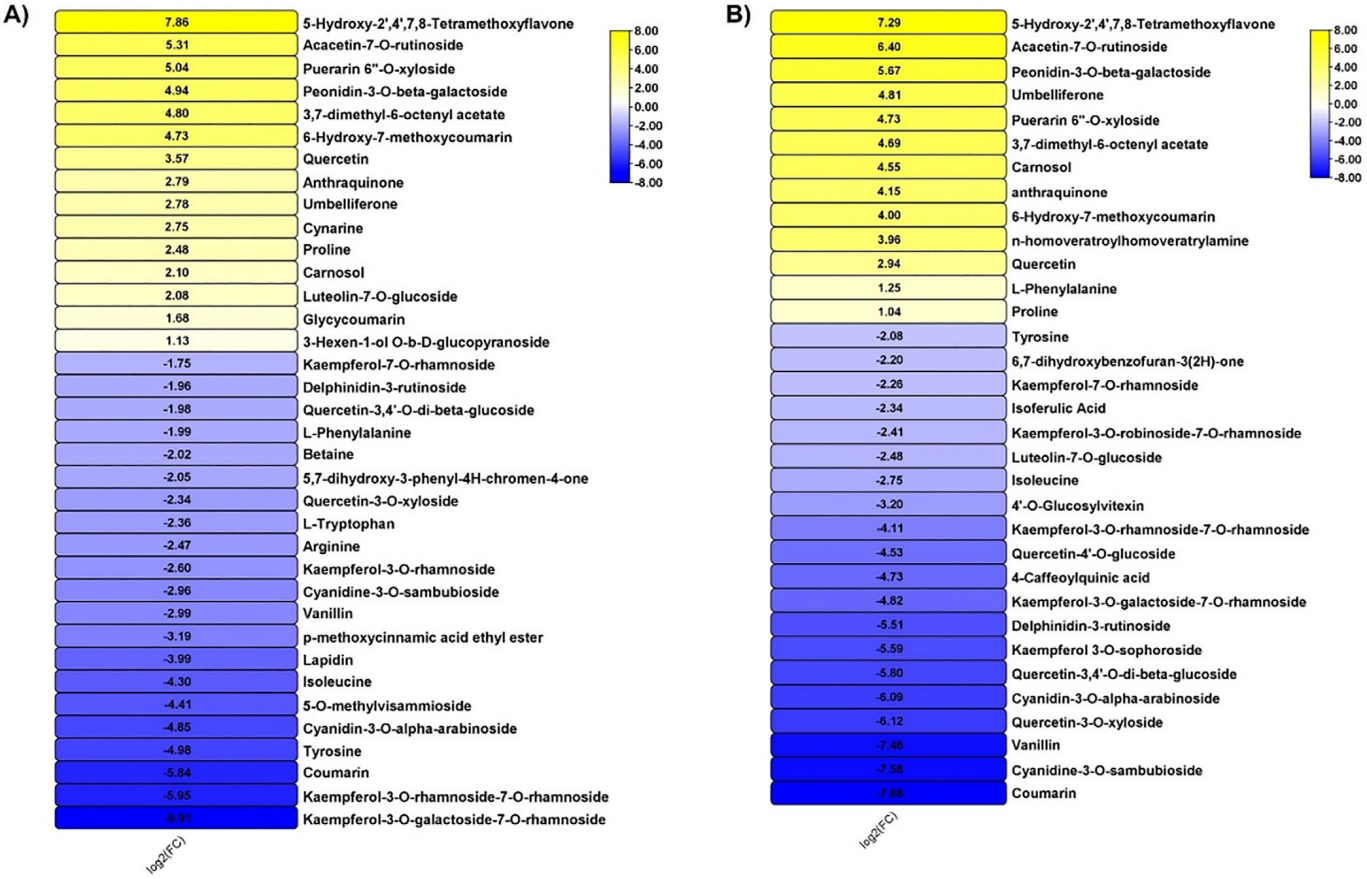
Heatmaps showing the log_2_ fold changes of metabolites detected in methanolic leaf waste extracts of floriculture crops. Yellow indicates metabolites with increased relative abundance (positive log_2_ fold change), whereas blue indicates metabolites with decreased relative abundance (negative log_2_ fold change). (A) Log_2_ fold changes of metabolites in *Chrysanthemum grandiflorum* relative to *Alstroemeria hybrida*. (B) Log_2_ fold changes of metabolites in *C. grandiflorum* relative to *Tagetes patula*.

### 3.4 Insecticidal Potential of Leaf Wastes From Different Floriculture Crops Against *Aphis craccivora*

#### 3.4.1 Virulence Against Nymphs of *A. craccivora*

Bioefficacy of prepared leaf waste extracts of *T*. *patula*, *C*. *grandiflorum*, and *A*. *hybrida* leaves showed significant mortality among nymphs of *A. craccivora* in a dose‐ and time‐dependent manner (Figure 3). Results showed that extracts of *C*. *grandiflorum* (4000 ppm) (Figure 3A), T*. patula* (10 000 ppm) (Figure 3B), and *A. hybrida* (25 000 ppm) (Figure 3C) resulted in comparable percent mortality to the recommended dose of Neembaan 96 HAT (Figure 3 and Table 3). Comparatively, *C*. *grandiflorum* leaf extract was most efficacious among the three studied extracts. The probit analysis showed that *C*. *grandiflorum* extract is highly effective (LC_50_ = 1398.36 ppm; LC_90_ = 5102.82 ppm) against *A. craccivora* nymphs, followed by *T*. *patula* (LC_50_ = 6693.42 ppm; LC_90_ = 14792.661 ppm) and *A*. *hybrida* (LC_50_ = 13921.41 ppm; LC_90_ = 27670.22 ppm) (Table 4). Additionally, lethal time analyses revealed that at higher concentrations, *C. grandiflorum* (4000 ppm) showed LT_50_ = 42.37 h, whereas, *T. patula* (10 000 ppm) and *A. hybrida* (25 000 ppm) showed LT_50_ values of 61.39 and 38.47 h, respectively (Table 5).

**FIGURE 3:**
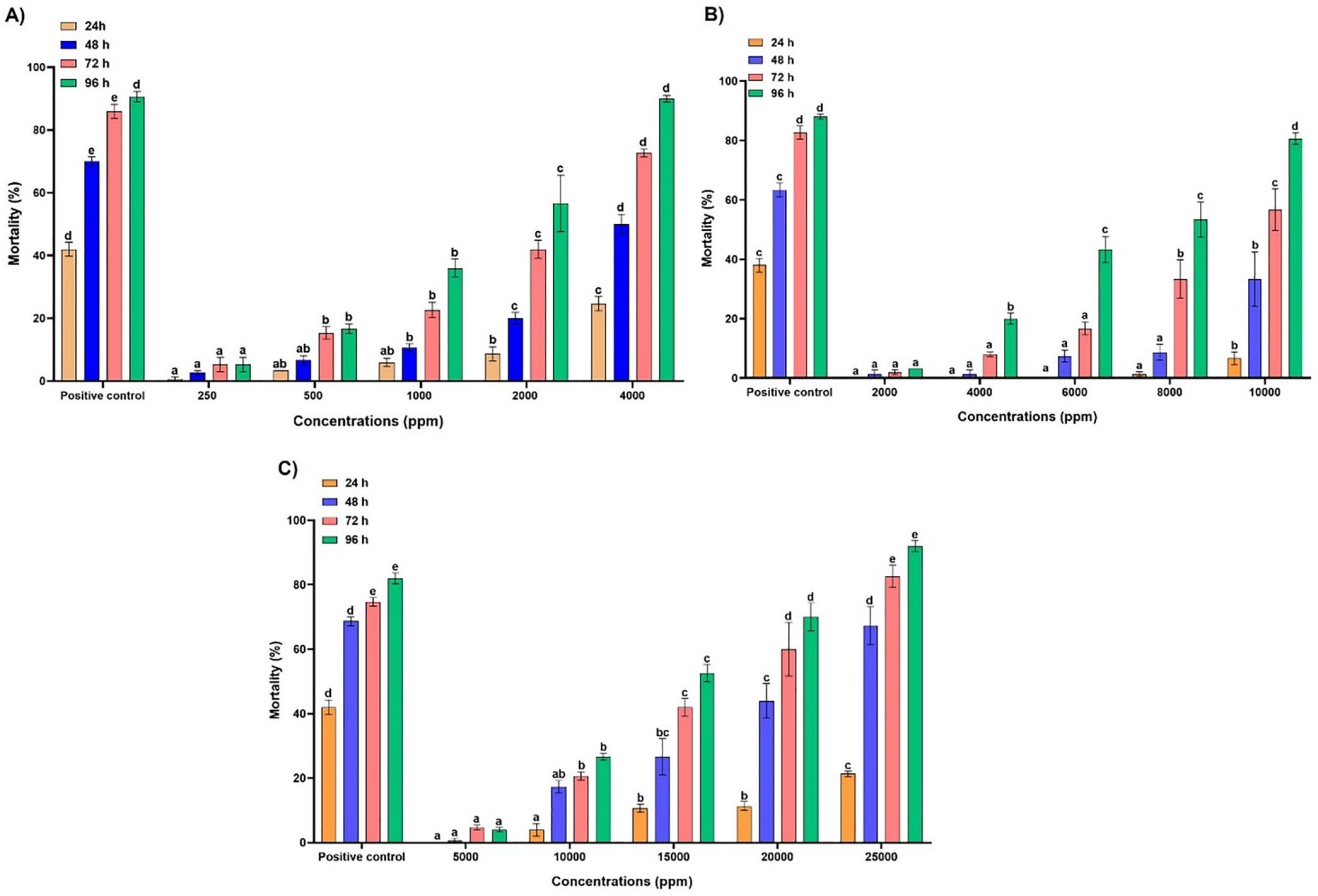
Percent mortality induced by methanolic extracts of leaf wastes of different floriculture crops against *Aphis craccivora* (nymph): (A) *Chrysanthemum grandiflorum*, (B) *Tagetes patula*, and (C) *Alstroemeria hybrida* at different concentrations. The bars represented the percent mean mortality. The same letters on bars do not differ significantly at *p* < 0.05 according to Duncan's test.

**TABLE 3:** Cumulative percent mortality of *Aphis craccivora* (% ± SE) after treated with different concentrations (ppm) of leaf wastes of floriculture crops 96 HAT.

Concentration (ppm)	Nymph (Mortality)	Adult (Mortality)	df	F	*p*‐value
*Chrysanthemum grandiflorum*					
250	5.33 ± 2.26^a^	25.33 ± 1.72^abc^	59	42.348	<0.001
500	16.67 ± 1.49^ab^	44.00 ± 7.10^cd^			
1000	36.00 ± 2.87^bcd^	59.33 ± 6.36^de^			
2000	56.67 ± 9.01^d^	80.67 ± 2.45^ef^			
4000	90.00 ± 1.05^f^	92.00 ± 5.01^f^			
Neembaan	90.67 ± 1.63^f^	92.00 ± 2.26^f^			
***Tagetes patula***					
2000	3.33 ± 0^a^	26.67 ± 6.91^bc^	59	60.962	<0.001
4000	20.00 ± 1.82^ab^	47.33 ± 4.14^de^			
6000	43.33 ± 4.35^cd^	63.33 ± 1.83^ef^			
8000	53.33 ± 5.87^de^	77.33 ± 2.87^fg^			
10 000	80.67 ± 1.94^fg^	86.67 ± 5.06^g^			
Neembaan	88.00 ± 0.82^g^	90.67 ± 2.45^g^			
***Alstroemeria hybrida***					
5000	4.00 ± 0.67^a^	30.67 ± 1.25^b^	59	208.047	<0.001
10 000	26.67 ± 1.05^b^	50.00 ± 1.83^c^			
15 000	52.67 ± 2.67^cd^	61.33 ± 2.26^de^			
20 000	70.00 ± 4.35^e^	82.00 ± 2.00^f^			
25 000	92.00 ± 1.70^fg^	98.67 ± 0.82^g^			
Neembaan	82.00 ± 1.70^f^	92.67 ± 1.94^g^			

Values represent the mean of % mortality ± standard error of five replicates (*n* = 150). Different letters within the column indicate statistically significant differences (*p* ≤ 0.05), as determined by Duncan's test.

*Abbreviations*: df, Degree of freedom

**TABLE 4:** Lethal concentrations (ppm) of leaf wastes of different floriculture crops against *Aphis craccivora* 96 HAT.

Plant	Target stage	LC_50_ (LL—UL) (ppm)	LC_90_ (LL—UL) (ppm)	Slope ± SE	χ^2^ value
*C. grandiflorum*	Nymph	1398.36 (1256.18−1565.05)	5102.82 (4207.41−6491.10)	2.28 ± 0.15	6.73
	Adult	637.49 (548.36−731.25)	3636.31 (2896.33−4879.33)	1.70 ± 0.13	1.15
*T. patula*	Nymph	6693.42 (6280.87−7156.56)	14792.66 (13031.35−17459.31)	3.72 ± 0.28	7.74
	Adult	3935.04 (3525.78−4329.81)	13308.62 (11296.24−16586.34)	2.42 ± 0.21	3.36
*A. hybrida*	Nymph	13921.41 (13128.09−14734.95)	27670.22 (25265.42−30980.86)	4.30 ± 0.29	7.49
	Adult	9096.80 (8128.75−10013.95)	29312.26 (25283.78−35628.53)	2.52 ± 0.21	2.70

Degree of freedom = 3

*Abbreviations*: LL, Lower limit; UL, Upper limit; ppm, Parts per million; LC, Lethal concentration; SE, Standard error of mean.

**TABLE 5:** Lethal time (hours) of leaf wastes of different floriculture crops against *Aphis craccivora*.

Plant	Target stage	Concentration (ppm)	LT_50_ (h)	LT_90_ (h)	Slope ± SE	χ^2^ value
*C. grandiflorum*	Nymph	250	1230.32 (334.70−36594996.05) *	10318.84 (1117.23−5.40 × 10^11^) *	1.39 ± 0.55	0.63
		500	375.00 (209.07−1736.03) *	2469.75 (770.94−55843.02) *	1.57 ± 0.37	1.22
		1000	155.50 (122.64−234.69) *	630.36 (367.53−1687.53) *	2.11 ± 0.32	3.22
		2000	86.80 (77.82 −100.09)	262.77 (200.54−395.18) *	2.66 ± 0.29	2.80
		4000	42.37 (38.51−46.13)	110.99 (96.92−132.89) *	3.06 ± 0.26	3.80
	Adult	250	405.90 (204.13−3199.92) *	5843.80 (1212.57−743596.04) *	1.10 ± 0.29	0.33
		500	128.01(95.98−230.34) *	1389.29 (554.21−11169.23) *	1.24 ± 0.25	0.70
		1000	72.05 (62.80−86.02)	373.22 ((245.27−763.54) *	0.25 ± 0.20	1.79
		2000	44.60 (39.14−49.97)	177.42 (138.94−257.19) *	2.14 ± 0.24	3.79
		4000	21.50 (16.46−25.76)	80.15 (68.65−99.81)	2.24 ± 0.27	0.91
*T. patula*	Nymph	2000	962.36 (267.36−1.98 ×10^25^) *	4840.75 (616.50 – 8.90 ×10^39^) *	1.83 ± 0.89	0.63
		4000	150.50 (124.57−224.63) *	296.17 (205.18−663.36) *	4.36 ± 0.84	0.09
		6000	107.92 (98.17−124.30) *	205.95 (167.21−289.09) *	4.57 ± 0.58	3.47
		8000	92.80 (85.56−102.23)	185.29 (155.14−238.65) *	4.27 ± 0.45	2.27
		10 000	61.39 (57.29−65.74)	131.62 (116.24−155.05) *	3.86 ± 0.31	2.15
	Adult	2000	223.65 (223.65 ‐154.84) *	1190.40 (536.16−6482.40) *	1.76 ± 0.33	1.40
		4000	120.06 (99.57−161.74) *	513.77 (320.13−1163.91) *	2.03 ± 0.28	4.89
		6000	75.43 (67.79−86.04)	252.99 (192.22−383.20) *	2.44 ± 0.27	2.69
		8000	61.06 (35.21−157.33)	215.73 (108.75−114582.78) *	3.03 ± 0.27	4.78
		10 000	37.19 (32.69−41.34)	119.21 (100.80−150.81) *	2.53 ±0.25	2.93
*A. hybrida*	Nymph	5000	709.95 (236.92 – 6.53×10^14^) *	2914.47 (497.427 – 9.08×10^14^) *	2.09 ± 0.99	0.30
		10 000	210.81 (147.30−441.28) *	1196.39 (533.89−6716.64) *	1.70 ± 0.32	2.43
		15 000	89.82 (78.64−108.04)	343.76 (240.55−610.52) *	2.20 ± 0.27	0.07
		20 000	59.24 (54.26−64.87)	167.45 (139.02−217.53) *	2.84 ± 0.27	2.06
		25 000	38.47 (35.20−41.59)	86.55 (78.11−98.45)	3.64 ± 0.28	1.29
	Adult	5000	385.19 (170.48−17985.98) *	20045.32 (1891.04‐ 2118848062) *	0.75 ± 0.2	0.73
		10 000	101.19 (82.34−143.30) *	745.21(387.27−2680.31) *	1.48 ± 0.25	1.28
		15 000	63.14 (53.59−77.06)	491.35 (282.10−1422.66) *	1.43 ± 0.24	0.87
		20 000	45.38 (40.33−50.41)	161.37 (130.14−221.24) *	2.32 ± 0.25	4.10
		25 000	31.97 (28.95−34.78)	70.21 (63.76−79.12)	3.75 ± 0.29	3.85

Degree of freedom 2; Values marked with (*) exceed the 96‐h experimental observation window.

*Abbreviations*: LL, Lower limit; UL, Upper limit; ppm, Parts per million; LT, Lethal time.

#### 3.4.2 Virulence Against Adults of *A. craccivora*

Bioefficacy studies of prepared leaf waste extracts of *T*. *patula*, *C*. *grandiflorum*, and *A*. *hybrida* showed significant mortality among adults of *A. craccivora* in a dose‐ and time‐dependent manner (Figure 4). Results showed that extracts of *C*. *grandiflorum* (2000 and 4000 ppm) (Figure 4A), *T. patula* (8000 and 10 000 ppm) (Figure 4B), and *A. hybrida* (25 000 ppm) (Figure 4C) resulted in comparable percent mortality to the recommended dose of Neembaan 96 HAT (Figure 4 and Table 3). Comparatively, *C*. *grandiflorum* extract was the most efficacious among the three studied extracts, showing the highest mortality. Additionally, the probit analysis also showed that *C*. *grandiflorum* extract was highly effective (LC_50_ = 637.49 ppm; LC_90_ = 3636.30 ppm) against *A. craccivora* adults, followed by *T*. *patula* (LC_50_ = 3935.04 ppm; LC_90_ = 13308.62 ppm) and *A*. *hybrida* (LC_50_ = 9096.80 ppm; LC_90_ = 29312.26 ppm) (Table 4). Additionally, lethal time analyses revealed that, at higher concentrations, *C. grandiflorum* (4000 ppm) showed LT_50_ = 21.50 h, whereas, *T. patula* (10 000 ppm) and *A. hybrida* (25 000 ppm) showed LT_50_ values of 37.19 and 31.97 h, respectively (Table 5).

**FIGURE 4:**
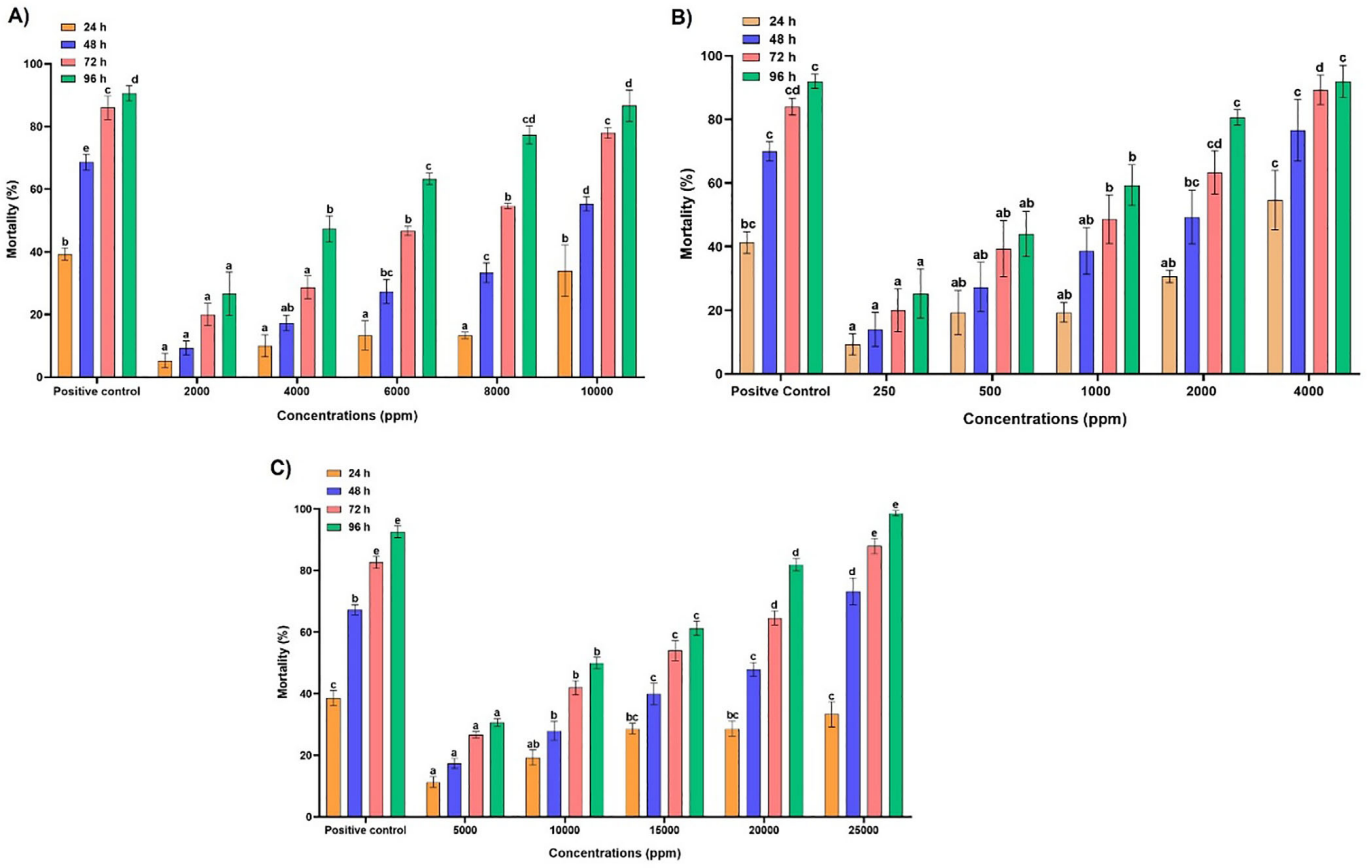
Percent mortality induced by methanolic extracts of leaf wastes of different floriculture crops against *Aphis craccivora* (adult): (A) *Chrysanthemum grandiflorum*, (B) *Tagetes patula*, and (C) *Alstroemeria hybrida* at different concentrations. The bars represented the percent mean mortality. The same letters on bars do not differ significantly at *p* < 0.05 according to Duncan's test.

### 3.5 Correlation of Identified Polyphenols Using UPLC With Insecticidal Activity

Spearman's correlation analysis showed strong positive associations between identified polyphenols and insecticidal activities (Figure 5). Strong positive correlation was observed between percent mortality and protocatechuic acid (Table 6). Similarly, ferulic acid, gallic acid, and kaempferol also showed positive correlation with the percent mortality, suggesting potential synergistic contribution of these polyphenols to overall toxicity of the extracts. In contrary, a strong negative correlation was observed between percent mortality and luteolin, indicates potential antagonistic interaction. Similarly, caffeic acid also exhibited negative correlation with percent mortality, distinguishing it from primary active constituents (Figure 5 and Table 6).

**FIGURE 5:**
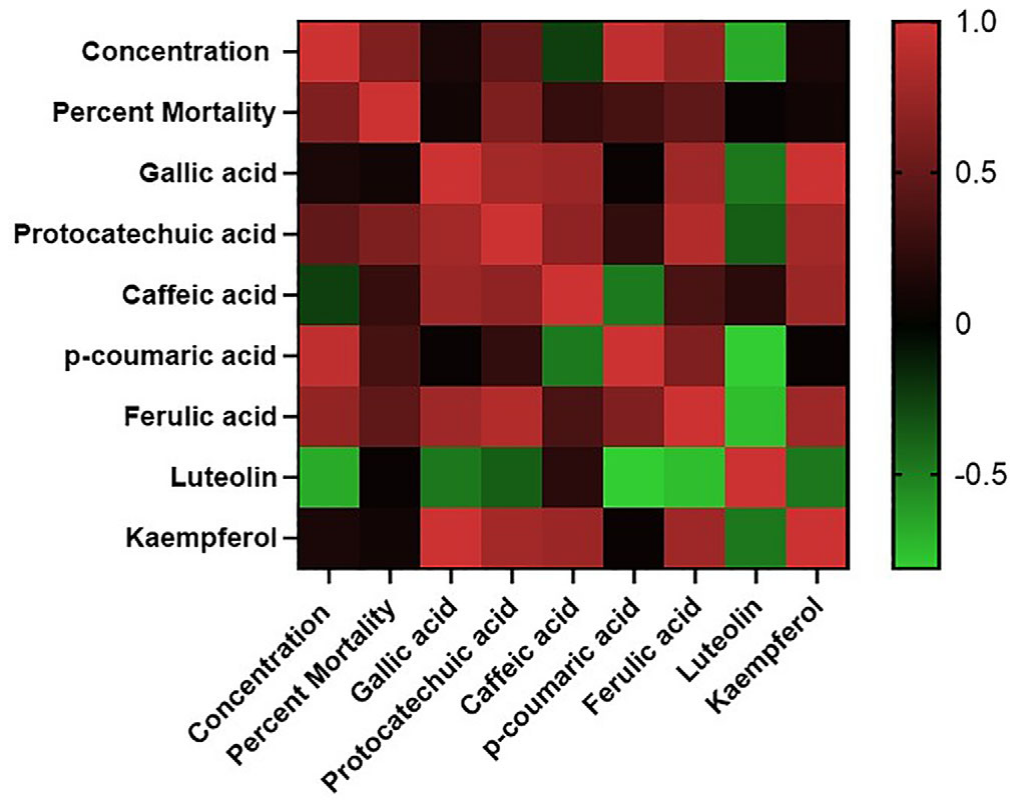
Spearman correlation analysis of extract concentrations, identified polyphenols, and insecticidal activity. The heat map illustrates the calculated Spearman rank correlation coefficients (*ρ*) calculated across all treatment groups (*n* = 15) for the extracts.

**TABLE 6:** Spearman's rank correlation coefficients (*ρ*) between treatment concentrations, percent mortality, and identified polyphenols.

Correlation coefficients (*ρ*)	Concentration	Percent mortality	Gallic acid	Protocatechuic acid	Caffeic acid	*p*‐coumaric acid	Ferulic acid	Luteolin	Kaempferol
Concentration	1	0.617728	0.122586	0.463744	−0.253449	0.935390	0.710835	−0.677955	0.122586
Percent mortality	0.617728	1	0.082922	0.610714	0.254588	0.334601	0.460714	0.044651	0.082922
Gallic acid	0.122586	0.082922	1	0.793079	0.747005	0.043305	0.769690	−0.474684	1.000000
Protocatechuic acid	0.463744	0.610714	0.793079	1	0.694660	0.240040	0.867857	−0.372088	0.793079
Caffeic acid	−0.253449	0.254588	0.747005	0.694660	1	−0.481481	0.360060	0.203532	0.747005
*p*‐Coumaric acid	0.935390	0.334601	0.043305	0.240040	−0.481481	1	0.614647	−0.811962	0.043305
Ferulic acid	0.710835	0.460714	0.769690	0.867857	0.360060	0.614647	1	−0.754807	0.769690
Luteolin	−0.677955	0.044651	−0.474684	−0.372088	0.203532	−0.811962	−0.754807	1	−0.474684
Kaempferol	0.122586	0.082922	1.000000	0.793079	0.747005	0.043305	0.769690	−0.474684	1

### 3.6 Detoxification Enzyme Activity Assay

The AChE and GST levels of *A. craccivora* exposed to LC_50_ concentrations of different treatments varied significantly with respect to treatment and negative control (*p ≤* 0.05). At 24 HAT, the AChE levels were significantly inhibited in case of positive control and *C. grandiflorum* extract, while in case of *T. patula* and *A. hybrida*, the AChE activity was significantly induced with respect to the negative control (Figure 6A). Similarly, at 48 HAT, the AChE activity of aphids exposed to the positive control and *C. grandiflorum* extract was significantly inhibited, while induced in case of *A. hybrida* and *T. patula* (Figure 6A).

**FIGURE 6:**
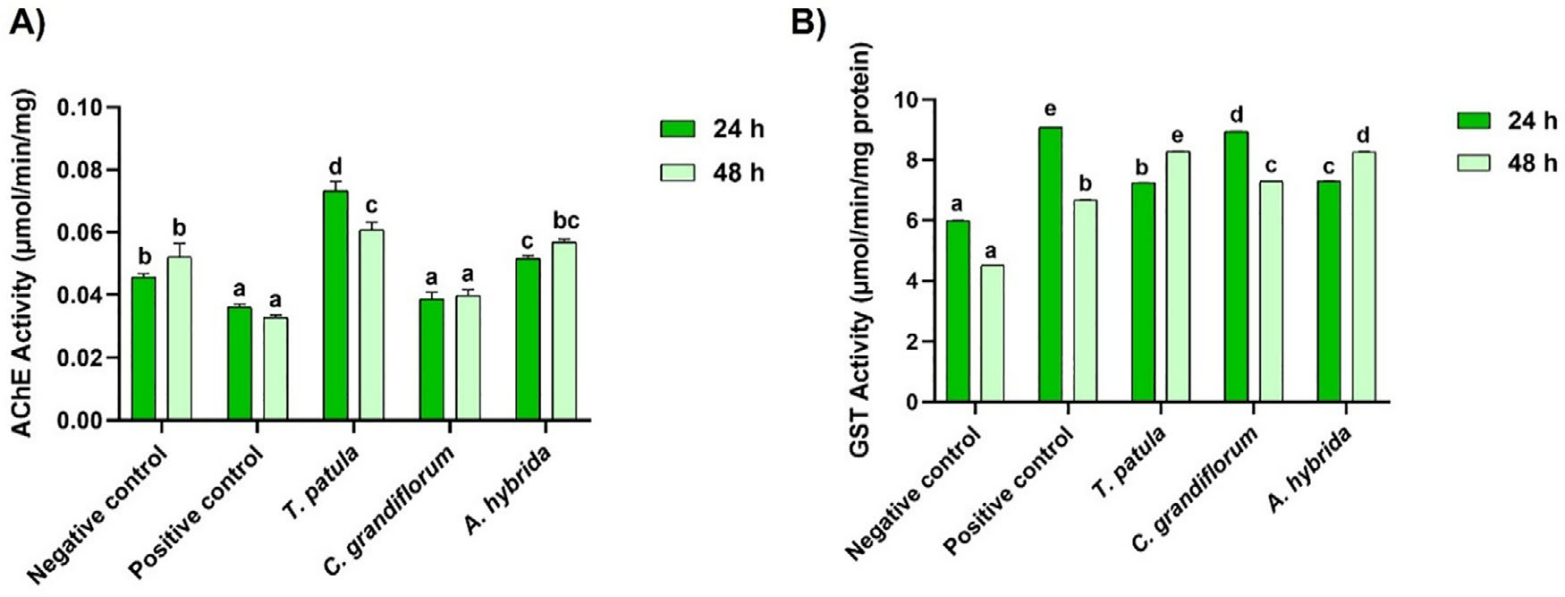
Enzyme activity of the methanolic extracts of leaf wastes of different floriculture crops against *Aphis craccivora*. (A) AChE and (B) GST. The same letters on bars do not differ significantly at *p* < 0.05 according to Duncan's test (*n* = 3).

In contrast, the levels of GST enzymes significantly increased as compare to the negative control at both 24 and 48 HAT (*p ≤* 0.05). At 24 h, the GST levels were significantly induced in all treatments with respect to the negative control, with the highest GST level observed in the positive control (9.084 ± 0.001 µmol/min/mL protein), followed by *C. grandiflorum* (8.948 ± 0.001 µmol/min/mL protein), *A. hybrida* (7.298 ± 0.004 µmol/min/mL protein), and *T. patula* (7.244 ± 0.007 µmol/min/mL protein), respectively (Figure 6B). By 48 h, a profound shift in GST levels were observed, the GST levels of aphids exposed to *T. patula* (8.289 ± 0.002 µmol/min/mL protein) and *A. hybrida* (8.266 ± 0.007 µmol/min/mL protein) surged significantly with respect to the positive control (6.665 ± 0.007 µmol/min/mL protein) and *C. grandiflorum* extract (7.296 ± 0.0001 µmol/min/mL protein), while the negative control exhibited the lowest levels (4.513 ± 0.001 µmol/min/mL protein) (Figure 6B). Overall, the statistical analyses indicate that both the levels and trend of AChE and GST enzyme activities were dependent on the type of treatment and the exposure duration.

## 4 Discussion

Floriculture has gained considerable acceptance in farmer communities in addition to the conventional crops due to its high market value. Nevertheless, postharvest flower production generates substantial quantities of unused leaves, resulting in considerable waste. These leaf wastes are often rich in metabolites of industrial significance. These metabolites are known to exhibit insecticidal potential and will be used to develop biopesticides from the waste [23]. This approach will help to solve two problems; one is waste management, and the other is reduced use of chemical insecticides, which ultimately prevent the environment. In literature, no studies have been reported on the insecticidal potential of the *C. grandiflorum*, *T. patula*, and *A. hybrida* leaf wastes against *A. craccivora*. Thus, the present study aimed to check the insecticidal potential of leaf wastes of selected floriculture crops against *A. craccivora*.

The present study showed that the virulence against *A. craccivora* depended upon the floriculture crops, concentrations, and the stage of insect pest. Methanolic extract of *C. grandiflorum* showed the highest aphicidal potential, followed by *T. patula* and *A. hybrida*. The extract of *C. grandiflorum* leaves has higher relative abundance of insecticidal metabolites, which corroborates with its highest insecticidal activity among other studied extracts. In particular, metabolites like acacetin‐7‐O‐rutinoside, quercetin [24], umbelliferone [24], carnosol [12], luteolin‐7‐O‐glucoside [25], and anthraquinone [26] are reported to possess insecticidal properties. The higher relative abundance of these metabolites may partly explain the stronger aphicidal activity of *C. grandiflorum*, although compound isolation and bioactivity‐guided fractionation are required to confirm their contribution. Moreover, the presence of high quantities of phenolics in the leaf waste extracts of floriculture crops might be the cause of the insecticidal activities. Additionally, concentrations of the extracts have a direct impact on the insect mortalities. It was observed that the mortality among the nymphs and adults of *A. craccivora* was in a dose‐dependent manner. Similarly, numerous studies have been reported to clearly demonstrate the dose‐dependent mortalities in *A. craccivora* when treated with plant extracts [11, 27]. Additionally, the life stage of the test insect is a crucial factor in insect mortalities. This study showed that adults of *A. craccivora* were more susceptible to all treatments than nymphs. Nymphs of *A. craccivora* have a waxy coating, irrespective of the adults, which might be the reason behind the lower toxicity of the extracts [28]. Furthermore, molting in nymphs may result in less absorption of extracts, which leads to lower mortality rates compared to adults. Behavioral changes, such as reduced feeding before and during molting, can help nymphs to withstand the insecticide applications.

Plant extracts enter the tissues and organs of target insects and alter the activity of several enzymes. Several secondary plant metabolites have the ability to inhibit enzyme activity, while others can increase the activity [29, 30, 31]. The activation or inhibition of several enzymes is linked to the lethal effects of secondary plant compounds on insects [32]. The LC_50_ concentrations were selected for the enzyme activity studies because they resulted in 50% surviving adults, which could be further used for analysis. While a study with higher concentrations would have resulted in a limited number of surviving aphids, and at lower concentrations less satisfactory enzyme activities would have been observed [33].

AChE is a serine hydrolase, vital to all species with cholinergic nerves [34]. Acetylcholine (ACh) is broken down into acetate and choline by the AChE during regular functioning, which terminates nerve impulses [35]. Secondary metabolites found in a number of botanical extracts prevent the synthesis of AChE, which causes ACh to build up in synapses and continuously stimulate nerve impulses [36]. This causes overexcitation, tremors, paralysis, and eventually the insect's death [12, 37]. In the present study the AChE activity depicted a distinctive pattern, inhibition in the case of the positive control and *C. grandiflorum* extract, while induction in the case of *T. patula* and *A. hybrida* extracts. This suggests that the unique phenolic compounds in the extracts guide the mode of action against the aphid's nervous system. The increase in AChE inhibition in the case of *C. grandiflorum* extract can be attributed to the presence of luteolin as an AChE inhibitor [38] or the combined action of luteolin along with protocatechuic acid and caffeic acid. While the induction of AChE activity in *T. patula* and *A. hybrida* extracts can probably be a survival strategy, a type of metabolic adaptation that enables the insects to produce excess enzyme to preserve their cholinergic nervous system in the presence of neurotoxins [39]. The observed increase or decrease in AChE activity may result from the combination of direct enzyme inhibition, compensatory enzyme regulation, detoxification responses, and selective survival of more tolerant individuals.

On the contrary, GST is a crucial enzyme and has a role in exogenous substance biotransformation, drug metabolism, and defense of the organism against oxidative damage [40]. It is known to catalyze the conjugation of electrophile molecules with reduced glutathione (GSH), wherein the potentially toxic substances become more water‐soluble and usually less toxic. In the present study, the induction of GST activity in aphids treated with botanical extracts indicates the stimulation of the detoxification system and a generalized physiological response [30]. The initial hike in GST activity in the positive control and *C. grandiflorum* at 24 h, followed by a decline at 48 h, suggests a transient induction, mediated by luteolin and caffeic acid. In contrast, the progressive increase in GST activity of aphids treated with *T. patula* and *A. hybrida* suggests sustained enzymatic induction, which may be linked to the chemical complexity and slower metabolism of their active constituents. Particularly, *T. patula* cause continuous oxidative pressure due to the presence of a high concentration of ferulic acid, which may act synergistically with gallic acid. Numerous studies have reported the role of detoxification enzymes, including GSTs, to metabolize deleterious plant secondary metabolites [41, 42, 43]. The relatively low GST activity in the negative control throughout the experiment reflects the basal detoxification levels in untreated insects. The observed differences among the extracts may be attributed to variations in phytochemical profiles, influencing their capacity to generate reactive metabolites that act as GST inducers. These findings imply that while all tested extracts activated detoxification pathways, their temporal patterns of GST induction differed, potentially affecting their long‐term efficacy against the target pest. The present study demonstrates that methanolic extracts from studied floriculture leaf wastes possess significant insecticidal activity against *A. craccivora* under laboratory conditions. These findings represent an initial proof of concept, and further research is required to evaluate field efficacy under diverse agroclimatic conditions, determine phytotoxicity, assess potential impacts on nontarget organisms, including natural enemies and pollinators, and develop cost‐effective extraction and large‐scale production strategies. Additionally, field transitions of these leads require novel formulations such as microencapsulation and eco‐friendly adjuvants to overcome environmental factors. These aspects will be essential to address to translate these promising laboratory findings into environmentally safe and commercially viable botanical insecticides.

## 5 Conclusions

This study showed that the leaf wastes of studied floriculture crops are a valuable yet underutilized source of insecticidal metabolites. The aphicidal bioassay results showed the highest insecticidal potential of leaf wastes of *C. grandiflorum*, followed by *T. patula* and *A. hybrida*. This study highlights the insecticidal potential of leaf wastes against *A. craccivora* and supporting their possible utilization as eco‐friendly alternatives to synthetic insecticides. The present study is relevant in the context of sustainable pest management and circular bioeconomy approaches by utilising leaf wastes of floriculture crops. Furthermore, the integration of targeted and nontargeted metabolomic approaches provided a comprehensive chemical profiling of the leaf waste extracts. Moreover, the presence of multiple bioactive metabolites suggests potential synergistic effects, which may enhance efficacy and reduce the risk of resistance development in insect populations. Overall, the findings underscore the promise of floriculture leaf wastes as sustainable raw materials for developing botanical insecticides. Future research should focus on the isolation of active compounds, evaluation under field conditions, assessment of impacts on nontarget organisms, and assessment of environmental safety.

## Author Contributions

**Aditya Singh Ranout**: methodology, investigation, validation, data curation, formal analysis, visualization, Writing – original draft preparation, writing – review and editing. **Rahul Kumar**: methodology, data curation, formal analysis, investigation, visualization, writing – original draft preparation, writing – review and editing. **Rupinder Kaur**: methodology, data curation, formal analysis, visualization, writing – original draft preparation, writing – review and editing. **Anish Kaachra**: visualization, formal analysis, data curation, writing – review and editing. **Anil Kumar**: formal analysis, data curation, writing – review and editing. **Dinesh Kumar**: Validation, writing – review and editing. **Bhavya Bhargava**: resources, writing – review and editing. **Gireesh Nadda**: conceptualization, funding acquisition, project administration, resources, supervision, validation, writing – review and editing. All authors read and approved the manuscript.

## Ethics Statement

Ethical approval was not required for this study, as it involved only a standard agricultural insect pest (*Aphis craccivora*). Invertebrate species are exempt from institutional and national animal welfare regulations.

## Conflicts of Interest

The authors declare no conflicts of interest.

## Supporting information




**Supporting File**: cbdv71720‐sup‐0001‐SuppMat.docx

## Data Availability

The data that supports the findings of this study are available in the Supporting Information of this article.
