# Supplementary material for: Aphicidal Potential and Metabolomic Profiling of Floriculture Leaf Waste Extracts Against Aphis craccivora Koch Under Laboratory Conditions
Source: Chem Biodivers. 2026 Sep 30;23(10):e71720. doi: 10.1002/cbdv.71720 (PMC13626257; doi:10.1002/cbdv.71720)
Supplement: Supplementary file 1 [file CBDV-23-e71720-s001.docx]

**Supplementary Data**

**Figure S1.** UPLC-PDA chromatograms of methanolic extracts of leaf wastes of different floriculture crops. A) Standard mixture; B) *Tagetes patula*; C) *Chrysanthemum grandiflorum*; D) *Alstroemeria hybrida*.

**Figure S2.** Data normalization comparison of data distribution before and after the normalization (median). TP: *Tagetes patula*; CG: *Chrysanthemum grandiflorum*; AH: *Alstroemeria hybrida*

**Table S1.** **UHPLC-QTOF-IMS based metabolite profiling of methanolic extracts of leaf wastes of floriculture crops**

| **Metabolite name** | **Average Rt (min)** | **Exact mass** | **MS/MS fragments** | **Ionization mode** | **Adduct type** | **Formula** | **Ontology** | **Total score** |
| --- | --- | --- | --- | --- | --- | --- | --- | --- |
| Proline | 1.968 | 115.06 | 116 | Positive | [M+H]^+^ | C_5_H_9_NO_2_ | Proline and derivatives | 86 |
| Betaine | 5.76 | 117.08 | 118 | Positive | [M+H]^+^ | C_5_H_11_NO_2_ | Alpha amino acids | 85.8 |
| Myrcene | 7.657 | 136.13 | 137 | Positive | [M+H]^+^ | C_10_H_16_ | Acyclic monoterpenoids | 82.3 |
| Lapidin | 5.565 | 334.21 | 335, 317 | Positive | [M+H]^+^ | C_20_H_30_O_4_ | Sesquiterpenoids | 90.8 |
| Indoline | 5.046 | 119.07 | 120, 103 | Positive | [M+H]^+^ | C_8_H_9_N | Indolines | 84.4 |
| Coumarin | 4.859 | 146.04 | 147, 119 | Positive | [M+H]^+^ | C_9_H_6_O_2_ | Coumarins and derivatives | 87.6 |
| Vanillin | 3.018 | 152.05 | 153, 135,125, 109, | Positive | [M+H]^+^ | C_8_H_8_O_3_ | Methoxyphenols | 87.8 |
| Arginine | 12.626 | 174.11 | 175 | Positive | [M+H]^+^ | C_6_H_14_N_4_O_2_ | L-alpha-amino acids | 82.2 |
| Tyrosine | 4.246 | 181.07 | 182, 165, 136 | Positive | [M+H]^+^ | C_9_H_11_NO_3_ | Tyrosine and derivatives | 91.8 |
| Harmalol | 17 | 200.09 | 201, 160 | Positive | [M+H]^+^ | C_12_H_12_N_2_O | Harmala alkaloids | 82.2 |
| Curdione | 19.673 | 236.18 | 259, 241 | Positive | [M+Na]^+^ | C_15_H_24_O_2_ | Germacrane sesquiterpenoids | 84.1 |
| Cyanidin | 16.913 | 287.24 | 273 | Positive | [M+H]^+^ | [C_15_H_11_O_6_]+ | 7-hydroxyflavonoids | 86.2 |
| Cafestol | 16.553 | 316.44 | 317, 299, 281 | Positive | [M+H]^+^ | C_20_H_28_O_3_ | Naphthofurans | 81 |
| Carnosol | 11.172 | 330.18 | 331, 287 | Positive | [M+H]^+^ | C_20_H_26_O_4_ | Diterpene lactones | 90.3 |
| Columbin | 1.806 | 358.14 | 381 | Positive | [M+Na]^+^ | C_20_H_22_O_6_ | Naphthopyrans | 90 |
| Cynarine | 10.026 | 516.13 | 517, 499, 163 | Positive | [M+H]^+^ | C_25_H_24_O_12_ | Quinic acids and derivatives | 88.1 |
| Allantoin | 14.921 | 158.04 | 181, 163, 135 | Positive | [M+Na]^+^ | C_4_H_6_N_4_O_3_ | Imidazoles | 91.7 |
| Harmaline | 2.335 | 214.11 | 215, 198 | Positive | [M+H]^+^ | C_13_H_14_N_2_O | Harmala alkaloids | 84.5 |
| Isoleucine | 2.388 | 131.09 | 132 | Positive | [M+H]^+^ | C6H13NO2 | Isoleucine and derivatives | 84.5 |
| Umbelliferone | 13.919 | 162.03 | 163, 145, 135, 119 | Positive | [M+H]^+^ | C_9_H_6_O_3_ | 7-hydroxycoumarins | 89.9 |
| Ascorbic acid | 7.92 | 176.03 | 199, 181 | Positive | [M+Na]^+^ | C_6_H_8_O_6_ | Butenolides | 87.5 |
| Anthraquinone | 8.126 | 208.05 | 231, 203, 147, | Positive | [M+Na]^+^ | C_14_H_8_O_2_ | Anthraquinones | 84.6 |
| Dihydrokawain | 10.701 | 232.11 | 233, 187, | Positive | [M+H]^+^ | C_14_H_16_O_3_ | Kavalactones | 86.5 |
| Columbianetin | 12.63 | 246.09 | 247, 229, 187, 147, | Positive | [M+H]^+^ | C_14_H_14_O_4_ | Angular furanocoumarins | 82.9 |
| Andrograpanin | 15.915 | 318.22 | 319 | Positive | [M+H]^+^ | C_20_H_30_O_3_ | Diterpene lactones | 87 |
| Glycycoumarin | 6.956 | 368.13 | 369 | Positive | [M+H]^+^ | C_21_H_20_O_6_ | Hydroxyisoflavonoids | 89.9 |
| Maoecrystal A | 6.958 | 388.19 | 389, 371, 355 | Positive | [M+H]^+^ | C_22_H_28_O_6_ | Kaurane diterpenoids | 82.5 |
| Secoxyloganin | 7.93 | 404.16 | 405, 243, 211, 193 | Positive | [M+H]^+^ | C_17_H_24_O_11_ | Terpene glycosides | 83.1 |
| Isokurarinone | 15.709 | 438.20 | 439 | Positive | [M+H]^+^ | C_26_H_30_O_6_ | 8-prenylated flavanones | 89.2 |
| Sciadopitysin | 10.36 | 580.14 | 581 | Positive | [M+H]^+^ | C_33_H_24_O_10_ | Biflavonoids and polyflavonoids | 88.3 |
| 3-Formylindole | 5.781 | 146.05 | 146, 118 | Positive | [M+H]^+^ | C_9_H_7_NO | Indoles | 92.9 |
| Ethyl caffeate | 12.014 | 208.07 | 209, 191, 175, 174 | Positive | [M+H]^+^ | C_11_H_12_O_4_ | Coumaric acids and derivatives | 86.1 |
| Linolenic acid | 16.496 | 278.22 | 279, 261, 243, 223 | Positive | [M+H]^+^ | C_18_H_30_O_2_ | Lineolic acids and derivatives | 88.4 |
| Isokaempferide | 11.841 | 300.06 | 301 | Positive | [M+H]^+^ | C_16_H_12_O_6_ | 3-O-methylated flavonoids | 91.9 |
| L-Phenylalanine | 3.383 | 165.08 | 166, 120, 103 | Positive | [M+H]^+^ | C_9_H_11_NO_2_ | Phenylalanine and derivatives | 93 |
| Isoferulic Acid | 11.994 | 194.06 | 195, 177 | Positive | [M+H]^+^ | C_10_H_10_O_4_ | Hydroxycinnamic acids | 86.3 |
| (+)-Costunolide | 12.954 | 232.15 | 233, 215, 187, 145, 131 | Positive | [M+H]^+^ | C_15_H_20_O_2_ | Germacranolides and derivatives | 86.8 |
| Ancistrocladine | 7.466 | 407.17 | 408, 393 | Positive | [M+H]^+^ | C_25_H_29_NO_4_ | Naphthylisoquinolines | 83.3 |
| Gentisyl alcohol | 11.347 | 140.05 | 141, | Positive | [M+H]^+^ | C_7_H_8_O_3_ | Hydroquinones | 86.5 |
| p-Coumaraldehyde | 22.991 | 148.05 | 149, 121 | Positive | [M+H]^+^ | C_9_H_8_O_2_ | Cinnamaldehydes | 86.2 |
| Methyl jasmonate | 2.402 | 224.14 | 247 | Positive | [M+Na]^+^ | C_13_H_20_O_3_ | Jasmonic acids | 88.3 |
| Cimiracemoside D | 10.113 | 678.40 | 679 | Positive | [M+H]^+^ | C_37_H_58_O_11_ | Cucurbitacin glycosides | 80.6 |
| D-(+)-Glucosamine | 4.566 | 179.08 | 180 | Positive | [M+H]^+^ | C_6_H_13_NO_5_ | Hexoses | 80.9 |
| Demethoxyyangonin | 19.209 | 228.08 | 251 | Positive | [M+Na]^+^ | C_14_H_12_O_3_ | Kavalactones | 82.2 |
| Trans-Cinnamic acid | 16.787 | 148.05 | 149, 131 | Positive | [M+H]^+^ | C_9_H_8_O_2_ | Cinnamic acids | 90.5 |
| 6''-O-Acetylglycitin | 5.366 | 488.13 | 511, 285 | Positive | [M+Na]^+^ | C_24_H_24_O_11_ | Isoflavonoid O-glycosides | 82.5 |
| 4'-O-Glucosylvitexin | 9.874 | 594.16 | 617, 471 | Positive | [M+Na]^+^ | C_27_H_30_O_15_ | Flavonoid O-glycosides | 88.4 |
| 4-Caffeoylquinic acid | 7.794 | 354.10 | 355, 337, 181, 163 | Positive | [M+H]^+^ | C_16_H_18_O_9_ | Quinic acids and derivatives | 89.6 |
| Quercetin-3-O-xyloside | 8.849 | 434.08 | 435, 303 | Positive | [M+H]^+^ | C_20_H_18_O_11_ | Flavonoid-3-O-glycosides | 92 |
| Luteolin-7-O-glucoside | 12.316 | 448.10 | 449, 287, 153, | Positive | [M+H]^+^ | C_21_H_20_O_11_ | Flavonoid-7-O-glycosides | 96.5 |
| 5-O-methylvisammioside | 11.421 | 452.17 | 453, 291, 273 | Positive | [M+H]^+^ | C_22_H_28_O_10_ | Furanochromones | 91.1 |
| Puerarin 6''-O-xyloside | 8.66 | 548.15 | 549 | Positive | [M+H]^+^ | C_26_H_28_O_13_ | Isoflavonoid C-glycosides | 84.5 |
| Acacetin-7-O-rutinoside | 9.335 | 592.18 | 593 | Positive | [M+H]^+^ | C_28_H_32_O_14_ | Flavonoid-7-O-glycosides | 92.3 |
| Quercetin-4'-O-glucoside | 15.883 | 464.10 | 465, 303, 229 | Positive | [M+H]^+^ | C_21_H_20_O_12_ | Flavonoid O-glycosides | 92.4 |
| Delphinidin-3-rutinoside | 8.453 | 611.16 | 611, 449, 303 | Positive | [M]^+^ | [C_27_H_31_O_16_]+ | Anthocyanidin-3-O-glycosides | 89 |
| Kaempferol-7-O-rhamnoside | 10.191 | 432.11 | 433, 287 | Positive | [M+H]^+^ | C_21_H_20_O_10_ | Flavonoid-7-O-glycosides | 93.9 |
| Kaempferol-3-O-rhamnoside | 10.408 | 432.11 | 433, 287 | Positive | [M+H]^+^ | C_21_H_20_O_10_ | Flavonoid-3-O-glycosides | 90.2 |
| Cyanidine-3-O-sambubioside | 9.173 | 581.15 | 581, 287, | Positive | [M]^+^ | C_26_H_29_O_15_ | Anthocyanidin-3-O-glycosides | 89.7 |
| Kaempferol 3-O-sophoroside | 8.562 | 610.15 | 611, 433, 287 | Positive | [M+H]^+^ | C_27_H_30_O_16_ | Flavonoid-3-O-glycosides | 95.3 |
| 6-Hydroxy-7-methoxycoumarin | 14.904 | 192.04 | 215, 200, 185 | Positive | [M+Na]^+^ | C_10_H_8_O_4_ | Hydroxycoumarins | 85 |
| Peonidin-3-O-beta-galactoside | 10.816 | 463.12 | 464, 301, 286, 258 | Positive | [M+H]^+^ | C_22_H_22_O_11_ | Anthocyanidin-3-O-glycosides | 93.7 |
| 2-hydroxynaphthalene-1,4-dione | 8.047 | 174.03 | 197 | Positive | [M+Na]^+^ | C_10_H_6_O_3_ | Naphthoquinones | 84.1 |
| 3,7-dimethyl-6-octenyl acetate | 8.265 | 198.16 | 219 | Positive | [M+H]^+^ | C_12_H_22_O_2_ | Fatty alcohol esters | 86.1 |
| Cyanidin-3-O-alpha-arabinoside | 9.982 | 419.10 | 419, 287 | Positive | [M]^+^ | [C_20_H_19_O_10_]+ | Flavonoid-3-O-glycosides | 93.1 |
| n-homoveratroyl homoveratrylamine | 9.584 | 345.19 | 382 | Positive | [M+Na]^+^ | C_20_H_25_NO_5_ | Dimethoxybenzenes | 88.3 |
| 6,7-dihydroxybenzofuran-3(2H)-one | 18.226 | 194.08 | 195 | Positive | [M+H]^+^ | C_10_H_10_O_4_ | Hydroxycinnamic acids | 87.7 |
| trans-4-Hydroxy-3-methoxycinnamate | 20.579 | 193.17 | 194, 177 | Positive | [M+H]^+^ | C_10_H_10_O_4_ | Hydroxycinnamic acids | 87.7 |
| p-methoxycinnamic acid ethyl ester | 7.736 | 206.09 | 207, 161 | Positive | [M+H]^+^ | C_12_H_14_O_3_ | Cinnamic acid esters | 80.5 |
| 3-Hexen-1-ol O-b-D-glucopyranoside | 9.773 | 262.14 | 263 | Positive | [M+H]^+^ | C_12_H_22_O_6_ | Fatty acyl glycosides of mono- and disaccharides | 85.2 |
| Quercetin-3,4'-O-di-beta-glucoside | 9.637 | 626.15 | 627, 465, 303 | Positive | [M+H]^+^ | C_27_H_30_O_17_ | Flavonoid-3-O-glycosides | 88.7 |
| 5,7-dihydroxy-3-phenyl-4H-chromen-4-one | 10.426 | 254.06 | 287 | Positive | [M+Na]^+^ | C_15_H_10_O_4_ | Isoflavones | 85.8 |
| 5-Hydroxy-2',4',7,8-Tetramethoxyflavone | 15.093 | 358.11 | 359, 341, 326, 310 | Positive | [M+H]^+^ | C_19_H_18_O_7_ | 8-O-methylated flavonoids | 92.9 |
| Kaempferol-3-O-rhamnoside-7-O-rhamnoside | 8.968 | 578.16 | 579, 287, 433 | Positive | [M+H]^+^ | C_27_H_30_O_14_ | Flavonoid-7-O-glycosides | 93.8 |
| Kaempferol-3-O-robinoside-7-O-rhamnoside | 7.443 | 740.22 | 741, 595, 433, 287 | Positive | [M+H]^+^ | C_33_H_40_O_19_ | Flavonoid-7-O-glycosides | 93.9 |
| Kaempferol-3-O-galactoside-7-O-rhamnoside | 8.417 | 594.16 | 595, 433, 287 | Positive | [M+H]^+^ | C_27_H_30_O_15_ | Flavonoid-7-O-glycosides | 95.5 |
| L-Tryptophan | 5.781 | 204.09 | 205, 188, 146, 144, 118 | Positive | [M+H]^+^ | C_11_H_12_N_2_O_2_ | Indolyl carboxylic acids and derivatives | 94 |
